# Supplementary material for: Deletion of wfs1 Impairs Oligodendrocyte Precursor Cells Dorsal Distribution and Myelination Through the wfs1-hmgcs1 Axis in Zebrafish
Source: Biology (Basel). 2026 Aug 16;15(16):1402. doi: 10.3390/biology15161402 (PMC13510151; doi:10.3390/biology15161402)
Supplement: Supplementary file 1 [file biology-15-01402-s001.zip › File S1-Transcriptome sequencing data and differentially expressed genes (DEGs) in wfs1-- zebrafish at 5 dpf/2.KEGG/wfs1KOvsWT/ALL/src/dre04136.html]

dre04136


- dre:792689

- Down regulated genes

792689\_1(-1.78857482493481)

- dre:406843

- Up regulated genes

406843\_1(1.98494944295698)

- dre:100000242

- Down regulated genes

100000242(-6.34512572021214)
- dre:326974

- Up regulated genes

326974\_1(1.07837684837292)

- dre:100000242

- Down regulated genes

100000242(-6.34512572021214)
- dre:326974

- Up regulated genes

326974\_1(1.07837684837292)

Close
